# Personal

**Published:** 1897-12

**Authors:** 


### Personal.

Dr. John Milton Duff, of Pittsburg, has been appointed gynecologist to the Western Pennsylvania Hospital. This has necessitated his resignation as gynecologist to the South Side Hospital, but he will continue to serve that institution as consulting surgeon and gynecologist. Dr. Duff's abilities will find a wider range in his new field and the Western Pennsylvania Hospital is to be congratulated on securing his excellent services.

Dr. A. Walter Suiter, of Herkimer, has lately been appointed by the regents of the University a state medical examiner to fill the vacancy occasioned by the death of Dr. William C. Wey, of Elmira. This is an admirable selection and Dr. Suiter will be welcomed to his new duties with enthusiasm by all the members of the examining board.

Dr. R. Harvey Reed, of Columbus, O., was elected president of the American Academy of Railway Surgeons at its last annual meeting held at Chicago. Dr. Reed is one of the founders of the academy, has been editor of its transactions from the first and will bring ability, familiarity with the work and enthusiasm into the chair.

Dr. Roswell Park is lately reported to have been in Italy and to be much improving in health. His many friends in Buffalo, as well as his pupils at the University, feel delighted at the prospect of his early return to his home recovered in health and ready to resume his professional and teaching labors.

Dr. Joseph Spangenthal, of Buffalo, has removed his office from 250 Masten street to 108 East Genesee street. Hours: 8–9 a. m., 2–4 and 7–8 p. m. Telephone, Bryant 646.

